# Complete genome sequence of *Sagittula stellata* strain E-37 reveals a plasmid-encoded type six secretion system

**DOI:** 10.1128/mra.00501-24

**Published:** 2024-10-22

**Authors:** Mary Nia M. Santos, John Raymond B. Salazar, Paul Christian T. Gloria, Alecia N. Septer

**Affiliations:** 1 Aquaculture Research and Development Division, National Fisheries Research and Development Institute, Quezon City, Philippines; 2 The Graduate School, University of Santo Tomas, Manila, Philippines; 3 Natural Sciences Research Institute, University of the Philippines, Quezon City, Philippines; 4 Department of Earth, Marine and Environmental Sciences, University of North Carolina, Chapel Hill, North Carolina, USA; Montana State University, Bozeman, Montana, USA

**Keywords:** Roseobacter, type VI secretion system, marine bacterium

## Abstract

We announce the complete genome sequence of *Sagittula stellata* strain E-37. The hybrid assembly of long and short reads revealed one chromosome and four plasmids. Furthermore, the genome analysis showed that the plasmid-encoded type six secretion system is linked to plasmid replication genes that may be common to Roseobacters.

## ANNOUNCEMENT

Roseobacters are widely distributed in the marine environment, accounting for about 25% of all marine bacteria (1). Aquaculture applications of several strains were demonstrated to be safe and effective against fish pathogens (2). The strain E-37 is a promising biocontrol agent candidate because it is non-pathogenic, is genetically tractable, and can tolerate a wide range of salinity, pH, and temperature (3). Here, we conducted whole genome sequencing to better characterize E-37.

The E-37 strain was isolated from the coastal waters in Georgia, USA (3) and deposited at the American Type Culture Collection (ATCC 700073^T^). Since the E-37 genome is only available as a contig level assembly (GCA_000169415.1), Illumina and Oxford Nanopore Technologies (ONT) sequencing were used to assemble it into the complete chromosomal level. The E-37 was plated on Seawater Tryptone (SWT) agar from a frozen glycerol stock; then, a colony was grown in SWT broth and incubated overnight at 29°C for DNA extraction using Quick-DNA Fungal/Bacterial Miniprep Kit (Zymo Research). Illumina sequencing libraries were prepared using the tagmentation-based and PCR-based Illumina DNA Prep kit and custom IDT 10 bp unique dual indices (UDI) with a target insert size of 320 bp. No additional DNA fragmentation or size selection steps were performed. Illumina sequencing was performed on an Illumina NovaSeq X Plus, producing 2 × 151 bp paired end reads. Demultiplexing, quality control, and adapter trimming were performed with bcl-convert1v4.1.5 (4). The Illumina reads were quality-checked using FastQC v0.11.0 (5) and trimmed using Trimmomatic v0.36.6 (6).

The genomic DNA was extracted using ZymoBIOMICS DNA Miniprep Kit (Zymo Research) from the bacterial cell pellet prior to ONT sequencing. The library was constructed using the Ligation Sequencing Kit (SQK-LSK109) and Native Barcoding Expansion 96 (EXP-NBD196), followed by sequencing on a MinION with a flow cell R9.4.1 (FLO-MIN106D). Base calling was performed using Guppy v6.4.2 with the high-accuracy model. A total of 117,963 reads were generated and quality-trimmed using Porechop v0.2.4 (7). Both long- and short-read sequencing were performed at SeqCenter (Pittsburgh, PA).

Sequencing yielded 508.2 Mb and 606.9 Mb of raw reads from Illumina and ONT platforms, respectively. Unicycler v0.5.0 (8) was used for hybrid assembly, followed by Pilon v1.20.1 (9) for polishing. Unicycler searched for *dnaA* and *repA* alleles in each replicon using TBLASTN to rotate the genome. The genome consisted of one chromosome and four plasmids, all of which are circular contigs and have a total length of 5,290,610 bp and a GC content of 65%. Based on QUAST v5.2.0 (10), the genome yielded 648.1× coverage. The assembly was annotated using the NCBI Prokaryotic Genome Annotation Pipeline (PGAP) v.6.5 (11), which identified 5,257 coding sequences, 13 rRNA genes, 132 tRNA genes, and four ncRNA genes. BUSCO v5.4.5 (12) indicated that the assembly has a completeness score of 100%. The genome encoded type I, II, IV, and VI secretion systems with either complete or fragmented gene members. Interestingly, T6SS is encoded on a plasmid, and the cluster is adjacent to plasmid replication genes, supporting previous findings that T6SSs are prevalent on plasmids in alpha-proteobacteria (13).

## Data Availability

This genome project has been registered at NCBI under BioProject accession number PRJNA1109544. The genome is available at Genbank under accession number GCA_039724765.1. The raw reads have been deposited at the NCBI Sequence Read Archive under the accession numbers SRR28974803 for the ONT raw reads and SRR28974804 for the Illumina raw reads.
